# FAM172A supervises ER (endoplasmic reticulum) stress‐triggered autophagy in the epidural fibrosis process

**DOI:** 10.1002/jsp2.1203

**Published:** 2022-05-01

**Authors:** Yufeng Zheng, Dianzhong Zhang, Le Su, Yanhua Wen, Yucai Wang

**Affiliations:** ^1^ Department of Orthopaedic Surgery, Tangdu Hospital Air Force Medical University Xi'an China; ^2^ Department of Orthopaedic Surgery The Fourth People's Hospital of Zibo Zibo China

**Keywords:** autophagy, calcium flux, epidural fibrosis (EF), ER (endoplasmic reticulum) stress, FAM172A, fibroblast

## Abstract

**Backgrounds:**

Lumbar laminectomy is usually utilized for lumbar disc herniation (LDH), but also causes epidural fibrosis (EF) process associated with abnormal proliferation of fibroblasts. FAM172A is associated with ER stress and cell proliferation, but its mechanism was unclear, especially in the process of EF.

**Methods:**

Therefore, the regulation of FAM172A on the calcium flux and autophagy in fibroblasts were investigated by inducing ER stress with tunicamycin and upexpression or downexpression of FAM172A. The calcium flux was determined using Fluo‐3, and autophagy was examined with immunofluorescence or western blot for LC3, Beclin‐1, ATG‐5, and p62. Moreover, the apoptotic protein of Bax and Bcl‐2 was detected, too. Furthermore, the laminectomy model was constructed and then dealt with overexpression of FAM172A.

**Results:**

Tunicamycin‐induced endoplasmic reticulum (ER) stress and autophagy process in fibroblasts were associated with the calcium flux regulated by FAM172A, especially in EF cells. Besides, tunicamycin induced autophagy and suppressed cell apoptosis of fibroblasts. Furthermore, FAM72A repressed the proliferation of fibroblasts and the process of EF in the laminectomy model through the mediation of the autophagy process.

**Conclusions:**

Tunicamycin‐induced endoplasmic reticulum (ER) stress in fibroblasts was associated with calcium flux mediated by FAM172A. FAM72A participated in the autophagy regulation of fibroblasts and maybe the key interaction regulator of apoptosis and autophagy in fibroblasts, especially for epidural scar cells.

## Graphical abstract

Through interference with FAM172A suppressed its expression resulted in a evident increase of intracellular calcuim flux, and the epidural scar tissue cell line ST was more visible. On the contrary, over‐expression of FAM172A significantly prohibited intracellular calcuim flux, especially in the epidural scar tissue cell line ST.

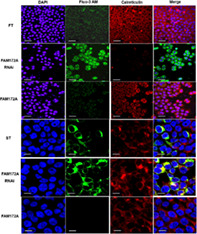

## 1 INTRODUCTION

Lumbar disc herniation (LDH) is the most common reason for back pain and is usually treated with lumbar laminectomy. But, lumbar laminectomy also causes a syndrome that comes mainly from epidural fibrosis (EF).^1^ EF maybe induced by abnormal proliferation of fibroblasts. Cellular homeostasis is major maintained by the catabolic process of autophagy,^2^ thereby it plays an important role in many pathologies, such as cell proliferation, neurodegeneration, and aging.^3^, ^4^ The formation of phagophore is the first step of autophagy,^5^ the membrane edges of phagophore prolong and then devour cytoplasm portions.^6^ Furtherly, the completed structure of autophagosome generates after edges fusion of membrane,^7^ then the formation of autolysosome combined autophagosome with lysosome.^8^ But, the recent mechanism of autophagosome biogenesis needs to be explored, especially in the process of EF.

The current studies confirm that the plasma membrane of different cellular compartments, including mitochondria, endoplasmic reticulum (ER), and Golgi may be associated with the precursors of autophagosome membrane.^9^ Moreover, the autophagic response can be induced by ER stress,^10^ which is caused by activation of unfolded protein response (UPR) come from misfolded proteins accumulation in ER.^11^ Meanwhile, the structure of the pre‐autophagosome assembles after stimulation of ER stress.^12^, ^13^ The previous experimental data demonstrated the biological functions of FAM172A; FAM172A was a stabilizing factor for ER stress. Moreover, FAM172A played a tumor suppressor role in hepatocellular carcinoma (HCC) under its supervision on calcium flux.^14^, ^15^

Besides, the death of cells could be regulated by two processes, including autophagy and apoptosis.^16^ The complex interactive regulation of the two processes between autophagy and apoptosis, which can be activated together through a variety of regulatory molecules and the stimuli of stress, even the two processes transform into each other.^17^

Therefore, the mechanistic investigation on the relationship between ER stress and autophagy associated with intracellular calcium flux mediated by FAM172A was studied in the progression of EF in the laminectomy model. Moreover, the regulation of FAM172A on the calcium flux and autophagy in fibroblasts were explored by inducing ER stress with tunicamycin, and up‐ or downexpression of FAM172A. The calcium flux was determined, and autophagy was examined based on the markers of autophagy.

## 2 MATERIALS AND METHODS

### 2.1 Animal model

The young male experimental rats (Sprague–Dawley, 250 ± 20 g) were purchased from the experimental animal center of Air Force Medical University (Xi'an, China). The experimental protocols were approved by the Animal Care and Research Committee of Air Force Medical University (Xi'an, China) according to the International Guide for the Care and Use of Laboratory Animals.

The ketamine (100 mg/kg body weight) was used to conduct anesthesia with intraperitoneal injection for the laminectomy model; the dura mater of the L1 vertebral plate was exposed. Then, the spinous process and vertebral plate of L1 were removed with rongeur forceps. To induce overexpression of FAM172A, the recombinant adenovirus vector was used to transfer the FAM172A gene, which was injected into the tail vein of the mice model.

At the end of the experiment, mice were over anesthetized with isoflurane (0.5 ml/min), until heartbeat and breathing stopped in the entire process but without pain. The unified approach was adopted to hand over animal carcasses.

### 2.2 Cell culture

Primary cells were directly isolated from living tissues of mices. In this experiment, fibroblasts were obtained from the living normal fiber tissues (FTs) of rat control (*n* = 6), and the living epidural scar tissues (STs) were isolated from rats which underwent laminectomies (*n* = 6), respectively. Then, these fibroblasts were cultured in vitro.

The obtained tissues were firstly cleaned with D‐Hanks and Hanks solution to remove blood stains on the surface of cells and the attached connective tissues. After a repeated clean process, a scalpel was used to cut the tissues into several small pieces about 1 mm^3^ in size, then was transferred into a small beaker contained with Hanks solution, Secondly, the above tissues were performed to digest and separate small pieces into cell clusters and scattered single cells with digestive enzymes, trypsin, and collagenase. Finally, the cell suspensions were incubated, and the cells were counted with a counting plate. The number of cells was adjusted to (2~5) × 10^5^ cells/ml with the culture medium, then cells were dealt with a CO_2_ incubator for 5% CO_2_, at 37°C. After 3~5 days, the primary cultured cells generally adhered to the wall of the bottle, then stretch and start to grow. Furthermore, the new medium was added into the original culture medium, then cells were continued to culture for 2~3 days, the medium was changed. At 7~14 days, the subcultured cells filled the wall of the bottle. The treatment with tunicamycin in the cell experiment was 48 h.

### 2.3 The plasmid transfections

The transfection plasmids were executed with jetPRIME agent (Polyplus‐transfection, Illkirch‐Graffenstaden, France) to achieve stable down‐ or overexpression of FAM172A.

The shRNA plasmids of the pSilencer 2.1‐neo vector were used to interfere the expression of FAM172A, and the RNAi‐targeting sequences for FAM172A were confirmed by the previous studies.^14^, ^15^

### 2.4 Western blot analysis

The protein electrophoresis was conducted with SDS‐PAGE (Sodium dodecyl sulfate‐polyacrylamide gel electrophoresis) gel. Moreover, blocked membrane transferred with proteins was dealt with skim milk and probed primary antibody. The primary antibodies were utilized in this work, such as β‐actin (13E5) (Cell Signaling; CST 4970S, dilution 1: 2000), FAM172A (Abcam; ab 121 364: dilution 1:2000), anti‐GRP78/BiP antibody (ab21685, 1:1000; Abcam); anti‐CHOP antibody (L63F7, CST2895, 1:800; Cell Signaling); anti‐LC3 (M152‐3: dilution 1:2000; MBL), anti‐Beclin‐1 (CST 3495: dilution 1:1500; Cell Signaling); anti‐ATG‐5 (CST 8540: dilution 1:1200; Cell Signaling); anti‐p62 (D5E2, CST 8025: dilution 1:1500; Cell Signaling); anti‐Bax (2D2, CST 89477: dilution 1:1000; Cell Signaling); anti‐Bcl‐2 (124, CST 15071: dilution 1:1500; Cell Signaling). Finally, the chemiluminescence examination was performed based on the secondary HRP antibody using exposed film.

### 2.5 Co‐localization

The cells were digested with trypsin; then, single‐cell suspensions were obtained. Moreover, the sterilized Petri dish was added a small amount of culture medium, then put the slide carefully into it, and drop the above single‐cell suspension onto the slide drop by drop. Finally, the dishes were placed in a 5% CO^2^ incubator at 37°C. After cultured for 48 h, the slides were washed three times with PBS at 37°C for 3–5 s each time. Subsequently, the cells on the surface of these slides were fixed in 4% paraformaldehyde for 15 min and rinsed with 37°C deionized water to clean the formaldehyde. Additionally, the process of antibody incubation was conducted for immunofluorescent staining. Finally, observe under the microscope of Zeiss 510 META microscope was used to observe the immunofluorescence in cells.

### 2.6 The determination of calcium flux

Cells were dealt with Fluo‐3 AM, which was the fluorescent probe for detecting calcium ion concentration in cells. The platform for life imaging service (Effingerstrasse 79, 4057 Basel, Switzerland) was utilized for this detection at 37°C. After washing with HBSS (Hanks Balanced Salt Solution) solution, the intensity and location of intracellular Fluo‐3 fluorescence were recorded and monitored at 340–380 nm.^17^ The concentration variation of free calcium in cells was represented by the ratio of F340/380 fluorescence intensity, which was the key factor for the cytosolic changes in Ca^2+^ concentrations.^18^ The image and fluorescence were obtained with the fluorescence microscope coupled to the camera, then analyzed using Fluorescence Ratio Imaging Software (version 7.0). A total of 50–100 cells were measured individually in each experiment group and were repeated three times.

### 2.7 Construction of recombinant adenovirus overexpression vector of FAM172A

The information of the CDS (Coding DNA Sequence) region of the target gene FAM172A was obtained from the NCBI website, then the primers were designed, and a high‐fidelity KOD enzyme (3 K mutation rate of 0%) was utilized to amplify from the template of cDNA library. Furthermore, it was cloned into the adenovirus system shuttle vector (Pshuttle‐CMV). The shuttle plasmid carrying the target gene fragment of FAM172A was linearized, and then, it was co‐transfected with the adenovirus plasmid into a specific E. coli for homologous recombination. The selected recombinant adenovirus was transfected into 293 cells by the liposome method (LipoFiter, Hanbio). Because the early gene E1 in the adenoviral vector genome was missing, the 293 cells with the E1 gene were used as packaging cells. The E1‐deleted adenovirus vector can be packaged in 2 weeks, and the virus particles can be enriched through multiple amplification. It can reach nearly 100% infection efficiency for most cell lines.

### 2.8 Epidural fibrosis assessment

For 4 weeks after surgery, the epidural scar tissues of surgical sites were evaluated according to the adhesion amount (Adhesion grade: 0, not adherent to dura mater; 1, adherent to dura mater, easily dissected; 2, adherent to dura mater, difficulty dissected without disrupting dura matter; 3, firmly adherent to dura mater, cannot be dissected.

### 2.9 Analysis of histology

Four weeks after surgery, the epidural scar tissues at the surgical sites were evaluated based on the histological analysis. The decalcified specimens of the whole L1 spinal column were fixed and sectioned. Finally, the stained sections with H&E (Hematoxylin–eosin) and Masson's trichrome were observed using light microscopy to evaluate the degree of epidural collagen tissues and EF.

Immunohistochemistry for GRP78 and CHOP proteins was performed using the avidin–biotin–peroxidase complex method. For antigen retrieval, the sections were treated with 0.5% trypsin at 37°C for 10 min. After blocking with 1% skim milk, tissue sections were incubated with anti‐GRP 78/BiP polyclonal antibody or anti‐CHOP antibody overnight at 4°C, and then washed in PBS solution. Biotinylated anti‐rabbit IgG was used as the secondary antibody, and the sections were visualized as previously described.

### 2.10 Statistics

The data were shown as mean ± SD (standard deviation). The differences between the two groups were analyzed with the Student's *t*‐test. Kruskal–Wallis ANOVA (Analysis of variance) was used to analyze the abnormally distributed data among groups. SPSS software (version 18.0) was utilized to perform these statistical analyses. *p* < 0.05 was significant statistically.

## 3 RESULTS

### 3.1 Tunicamycin‐induced endoplasmic reticulum (ER) stress in fibroblasts was associated with calcium flux regulated by FAM172A

**FIGURE 1:**
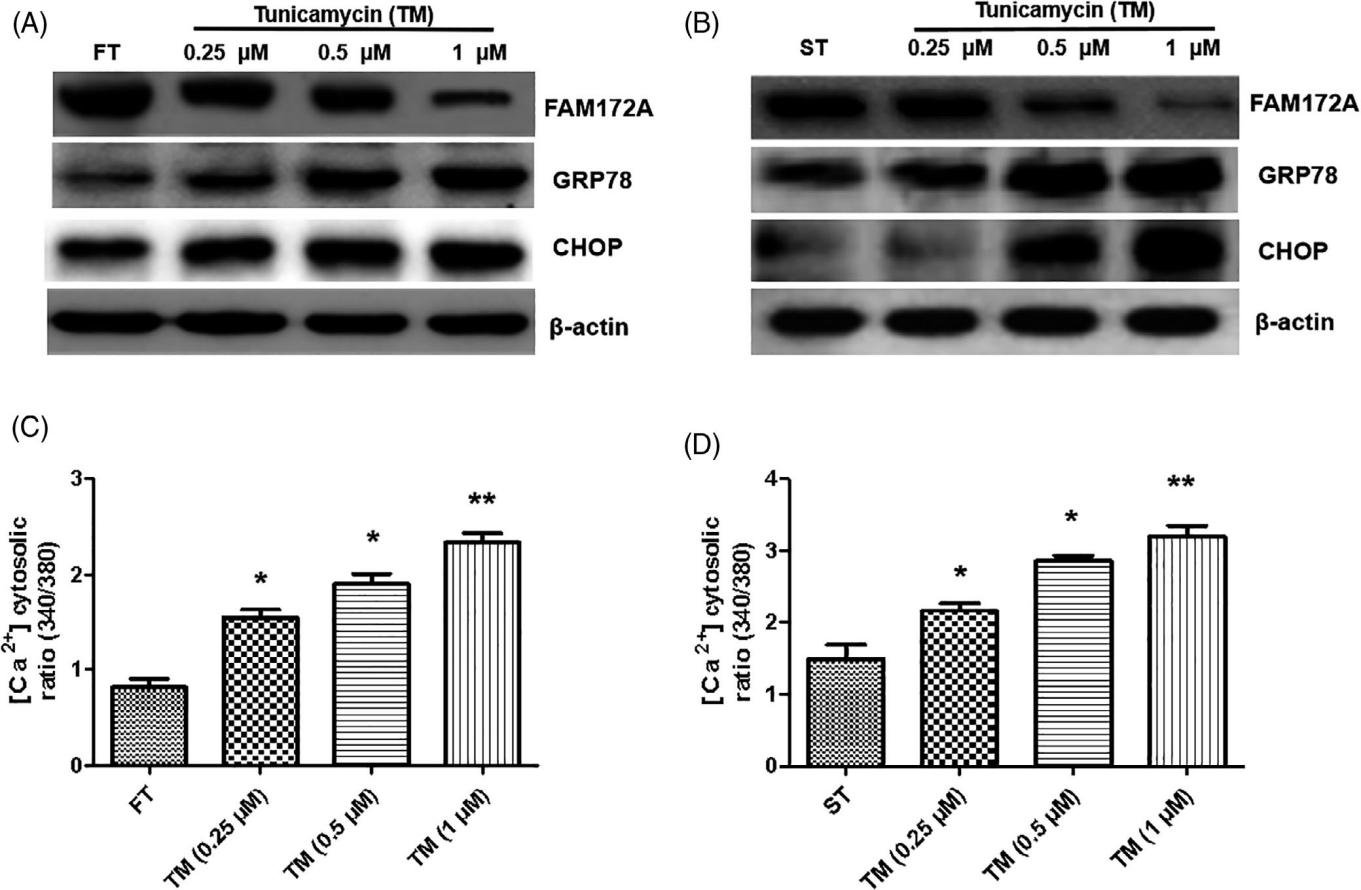
Tunicamycin‐induced process of the endoplasmic reticulum (ER) stress in fibroblasts was associated with the calcium flux regulated by FAM172A. After tunicamycin treatment, the expression of the endoplasmic reticulum stress marker protein GRP78, CHOP, and FAM172A was determined in both FT (A) and ST (B) cell lines. In addition, with the increase of the tunicamycin concentration, the intracellular calcium flux was both detected in FT (C) and ST (D). (The number of experimental replicates was three. *p < 0.05; **p < 0.01)

At first, we used the recognized tunicamycin to induce the endoplasmic reticulum (ER) stress in fibroblasts. After treatment with tunicamycin, FT (Fibrosis tissue) (Figure 1A) and ST (Epidural scar tissue) (Figure 1B) both evolved into the endoplasmic reticulum (ER) stress. The expression of endoplasmic reticulum stress marker protein GRP78 showed a dose‐dependent increase with the rising concentration of tunicamycin, especially in ST. On the contrary, the expression of FAM172A showed a dose‐dependent decrease with the stepwise tunicamycin concentration, and it was more significant in ST.

Our experimental data indicated that the key protein of the endoplasmic reticulum stress signaling pathway, CHOP,^19^, ^20^, ^21^ also increased in a dose‐dependent manner with the stepwise tunicamycin concentration, and its expression was more significant in FT (Figure 1A, B, and Figure 1A, B).

**FIGURE 2:**
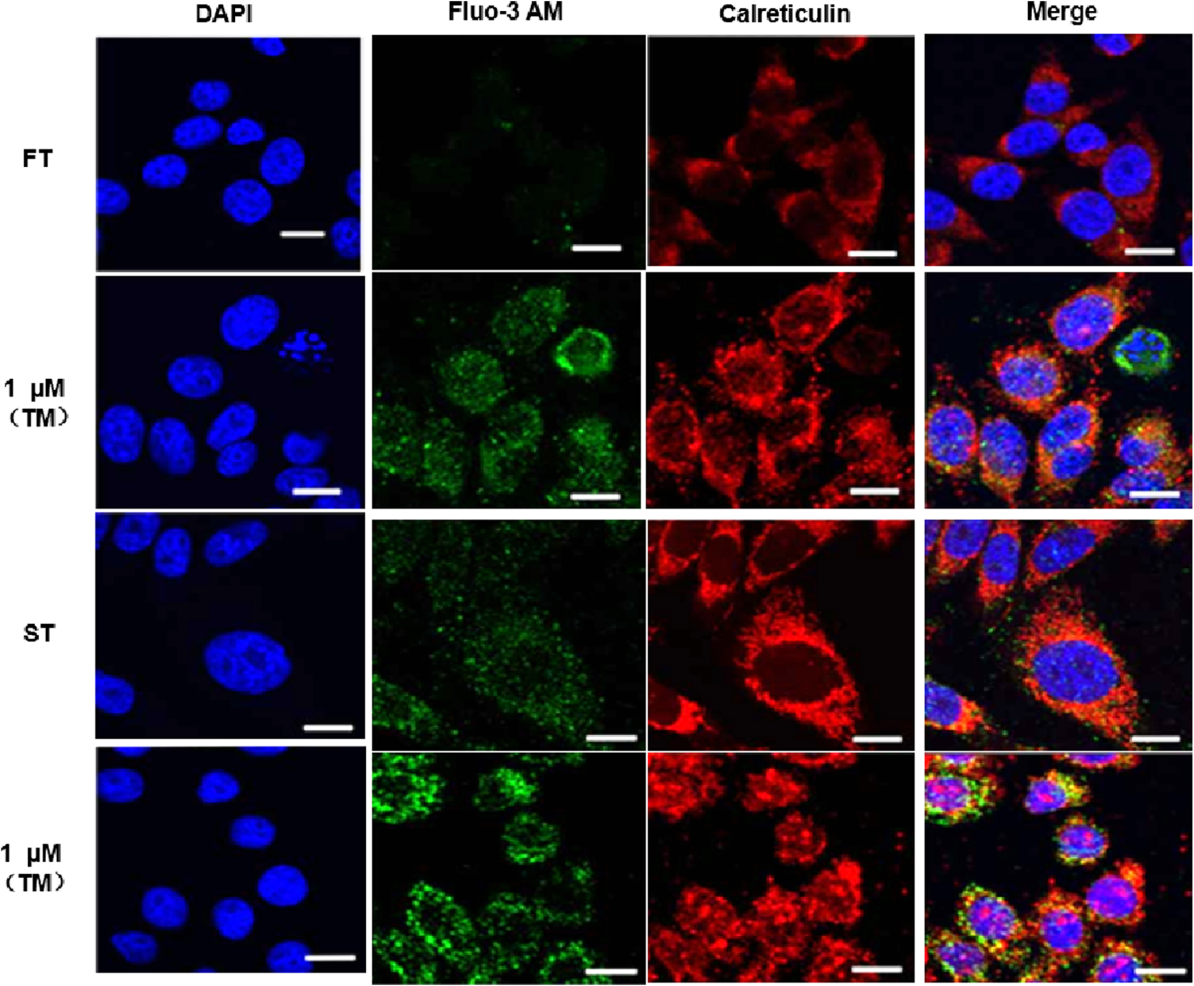
Tunicamycin‐induced endoplasmic reticulum (ER) stress in fibroblasts was related to the calcium flux regulated by FAM172A based on the experiment results of the confocal microscope. The confocal microscope was used to exhibit the calcium current in FT and ST cell lines after treatment with or without tunicamycin. (The number of experimental replicates was three. Scale bar is 1 μm)

In addition, the results of the intracellular calcium flux detection experiment identified that with the increased concentration of the tunicamycin, the intracellular calcium flux showed a dose‐dependent enhancement, and it was more marked in FT (Figure 1C and D). The laser confocal microscope was further used to detect the intracellular calcium current; calreticulin was used as an internal reference, which was located at the endoplasmic reticulum.^22^ It indicated that the calcium current in ST was distinctly stronger than that of the normal fibrosis tissue, meanwhile the calcium current was induced by tunicamycin in the two cell lines which were both significantly increased, and the calcium flux of ST enhanced more visibly (Figure 2).

### 3.2 FAM72A participated in the regulation of the endoplasmic reticulum stress‐related calcium flux

**FIGURE 3:**
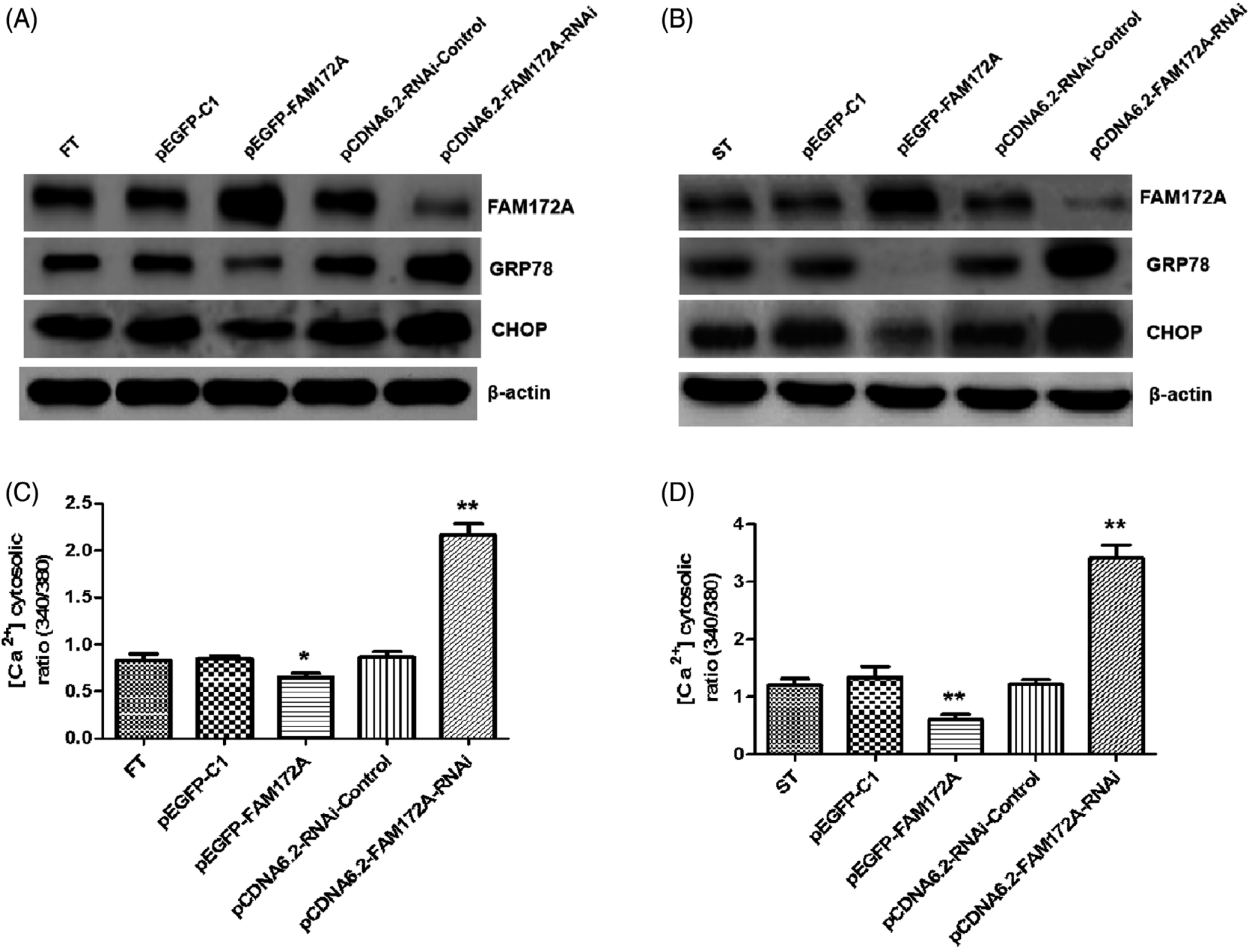
FAM72A participated in the regulation of the endoplasmic reticulum stress‐related calcium flux. Through overexpression and interference with the expression of FAM172A, it was aimed to clarify whether FAM172A regulates the process of endoplasmic reticulum stress in FT (A) and ST (B) by detecting the marker protein of the endoplasmic reticulum stress, GRP78, the key proteins CHOP, and FAM172A of the signal transduction pathway. Furthermore, the detection experiments of intracellular calcium flux were performed in FT (C) and ST (D) cell lines, respectively. (The number of experimental replicates was three. *p < 0.05; **p < 0.01)

Through overexpression and interference with the expression of FAM172A, it was aimed to clarify whether FAM172A regulated the process of the endoplasmic reticulum stress in FT (Figure 3A) and ST (Figure 3B). By examining the expression levels of the marker protein GRP78 of endoplasmic reticulum stress and the key protein CHOP in the signal transduction pathway of the ER stress,^23^ we found that the overexpression of FAM172A resulted in a decreased expression levels of GRP78 and CHOP in the two cell lines, and ST was more obviously. On the contrary, the interference with the expression of FAM172A caused significantly increased expression levels of GRP78 and CHOP in the two cell lines, and the epidural scar tissue cell line ST was more obvious (Figure 3A, B and Figure 3A, B).

**FIGURE 4:**
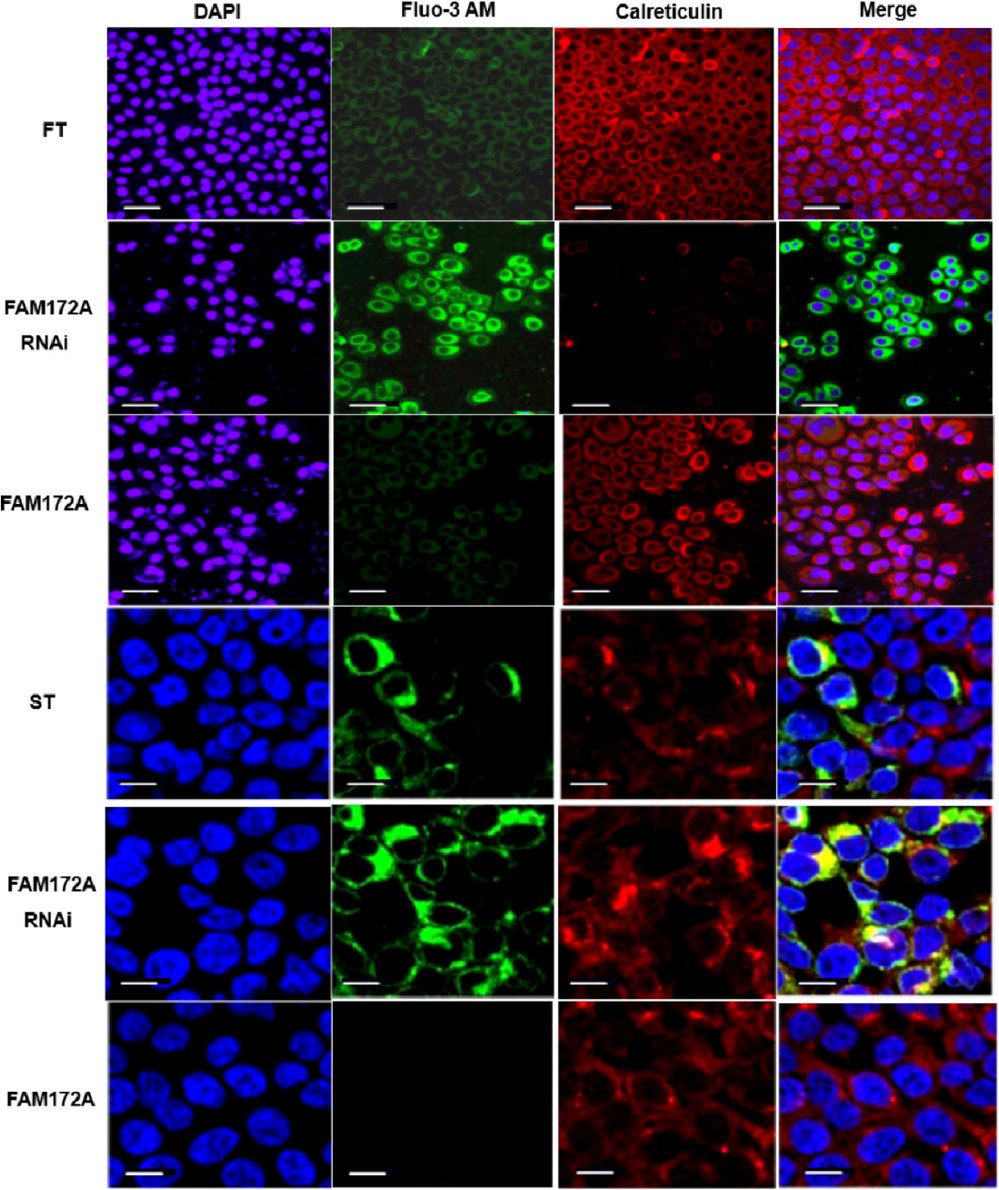
FAM172A participated in the regulation of the endoplasmic reticulum stress‐related calcium flux according to the laser confocal images. The laser confocal microscope was further used to detect the intracellular calcium flux, and calreticulin was used as an internal reference, which was located at the endoplasmic reticulum. Through overexpression and interference with the expression of FAM172A, it was aimed to clarify whether FAM172A regulated the endoplasmic reticulum stress process of FT and ST. (The number of experimental replicates was three. Scale bar was 5 μm in FT and 1 μm in ST.)

Furthermore, through intracellular calcium flux detection experiments, it was found that overexpression of FAM172A significantly inhibited intracellular calcium flux (*p* < 0.01), especially for ST (Figure 3C and D). On the contrary, restraining the expression through interfering with FAM172A resulted in a prominent promotion of intracellular calcium flux (*p* < 0.01), especially in ST (Figure 3C and D). The laser confocal microscope was further used to detect intracellular calcium flux, meanwhile calreticulin was used as an internal reference. The results suggested that interference with FAM172A suppressed its expression resulting in an evident increase of intracellular calcium flux (*p* < 0.01), and the epidural scar tissue cell line ST was more visible (Figure 4). On the contrary, overexpression of FAM172A significantly prohibited intracellular calcium flux (*p* < 0.01), especially in the epidural scar tissue cell line ST (Figure 4 and Figure 4).

### 3.3 Tunicamycin induced autophagy of cell lines and inhibited cell apoptosis regulated by FAM172A

**FIGURE 5:**
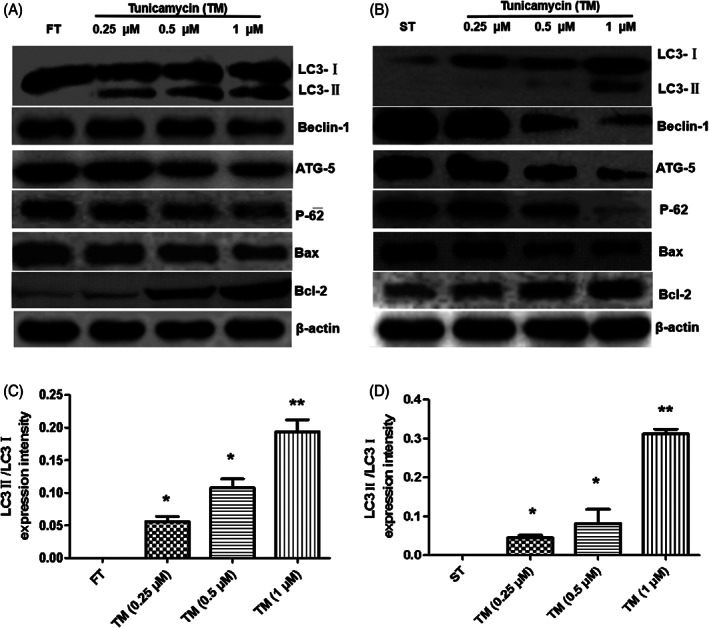
Tunicamycin induced autophagy and inhibited cell apoptosis regulated by FAM172A. After tunicamycin treatment, the autophagy marker protein LC3‐II expression, the expression of autophagy key regulatory protein Beclin‐1, ATG‐5, and autophagy protein p62 were all valuated with western blot experiments in both FT (A) and ST(B) cell lines, respectively. Furthermore, the detection experiments of intracellular calcium flux were performed in FT (C) and ST (D) cell lines, respectively. (The number of experimental replicates was three. *p < 0.05; **p < 0.01)

After tunicamycin treatment, both the two cell lines, FT (Figure 5A) and ST (Figure 5B) occurred autophagy (Figure 5A, B). In the meantime, the autophagy marker, LC3‐II protein expression,^24^ increased in a dose‐dependent manner followed with the stepwise tunicamycin concentration, and it was more significant in ST (Figure 5C, D). Besides, the expression of autophagy key regulatory protein Beclin‐1,^25^ ATG‐5,^26^ and autophagy protein p62^27^ all showed a dose‐dependent decline followed with the increased tunicamycin concentration, and the expression of these marker proteins in epidural scar tissue cell line ST was more obviously decreased. On the contrary, the expression of apoptosis‐promoting protein Bax of the Bcl‐2 family also declined in a dose‐dependent manner as the enhanced concentration of tunicamycin. Moreover, the expression of the apoptosis‐inhibiting protein Bcl‐2 increased significantly, and in the epidural scar tissue cell line, ST was more prominent (Figure 5A and B).

**FIGURE 6:**
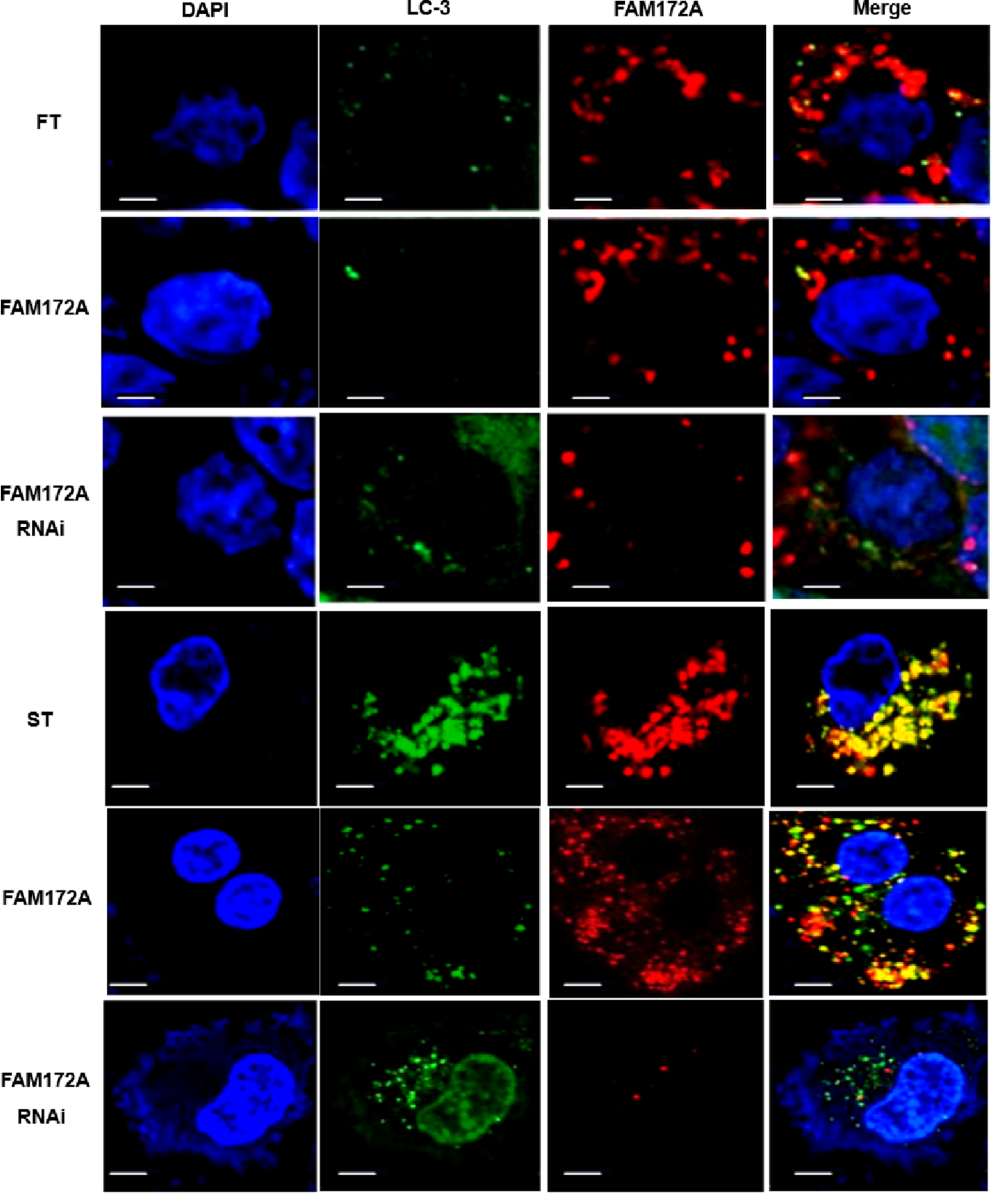
The confocal images indicated that tunicamycin induced autophagy of fibroblasts associated with FAM172A. Further application of laser confocal microscopy was used to detect the autophagy status that appeared in both FT and ST cell lines after treatment with tunicamycin. (The number of experimental replicates was three. Scale bar was 0.25 μm.)

Further application of laser confocal microscopy was used to detect the autophagy status of cells; the results suggested that after tunicamycin treatment, autophagy both appeared in cell lines FT and ST, and the autophagy status of ST was more visible (Figure 6). Moreover, tunicamycin induced downregulation of FAM172A expression in both cell lines (Figure 6).

### 3.4 FAM72A participated in the regulation apoptotic autophagy process of fibroblasts

**FIGURE 7:**
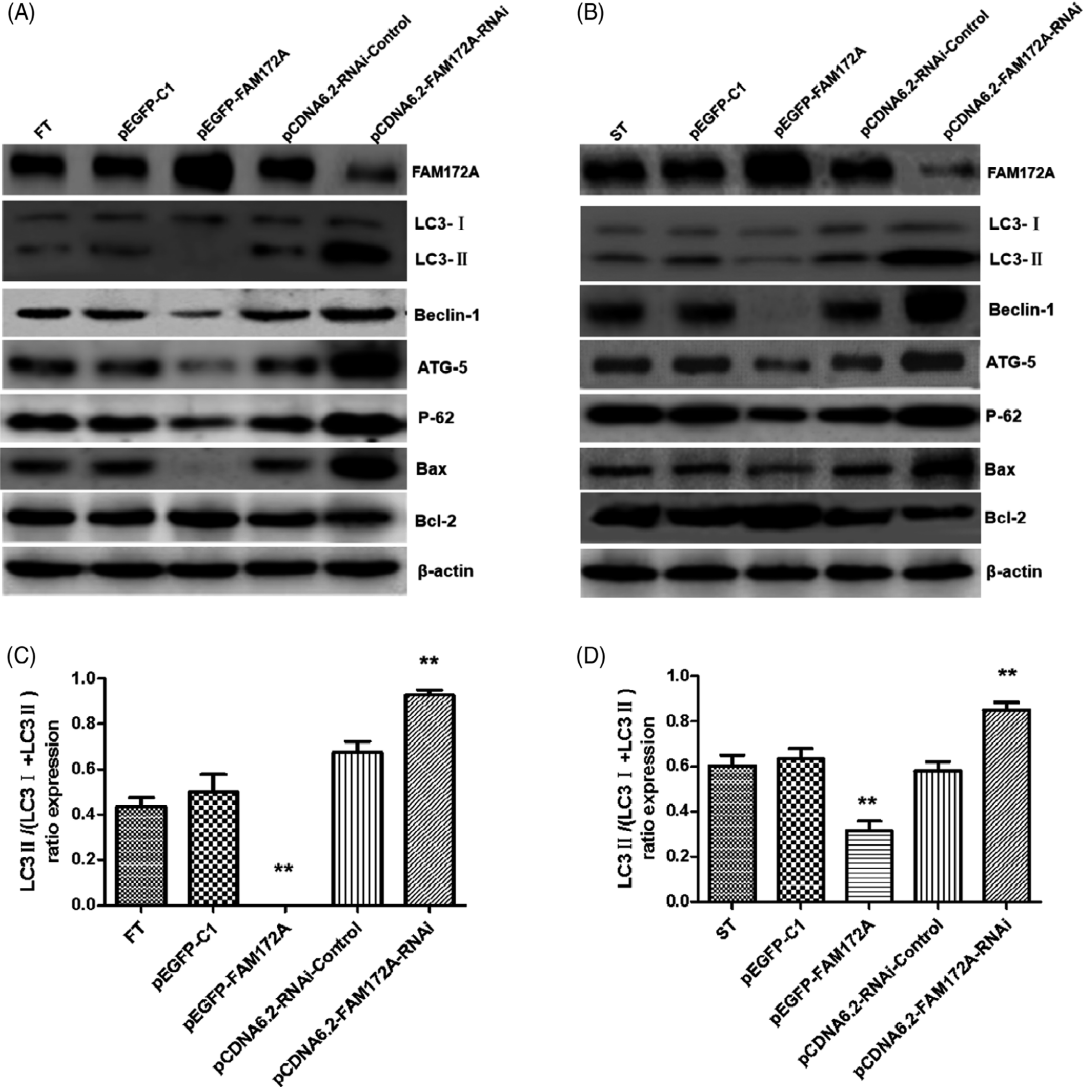
FAM72A participated in the regulation of fibroblasts autophagy. Through overexpression and interference with the expression of FAM172A, it was the purpose to confirm whether FAM172A regulates the autophagy process of FT (A) and ST (B) by detecting the expression levels of the autophagy marker protein LC‐3 and its subtypes LC3‐I, LC3‐II, and then the expression ratio of the autophagy marker protein LC3‐II in the two cell lines (C and D) was evaluated. (The number of experimental replicates was three. **p < 0.01)

Through overexpression and interference with the expression of FAM172A, the purpose was to confirm whether FAM172A regulated the autophagy process of FT (Figure 7A) and ST (Figure 7B). Through examining the autophagy marker protein LC‐3, especially for the expression levels of its subtypes LC‐3I and LC‐3II, we found that the overexpression of FAM172A resulted in a decreased expression ratio of the autophagy marker protein LC3‐II in the two cell lines; in the meantime, the epidural scar tissue cell line ST was more prominent. On the contrary, interference with the expression of FAM172A made for a visibly increased expression ratio of the autophagy marker protein LC3‐II in the two cell lines, while the normal fibrosis tissue cell line was more evident at this time (Figure 7A–D and Figure 7A, B).

Besides, the overexpression of FAM172A resulted in decreased expression of the key regulatory protein complexes of autophagy Beclin‐1, ATG‐5, and autophagy protein p62 in the two cell lines, and this was also more pronounced in ST. In addition, FAM172A overexpression inhibited the expression level of apoptosis‐promoting protein Bax and promoted the expression of apoptosis‐inhibiting protein Bcl‐2; this was more marked in the ST (Figures 7A and B).

**FIGURE 8:**
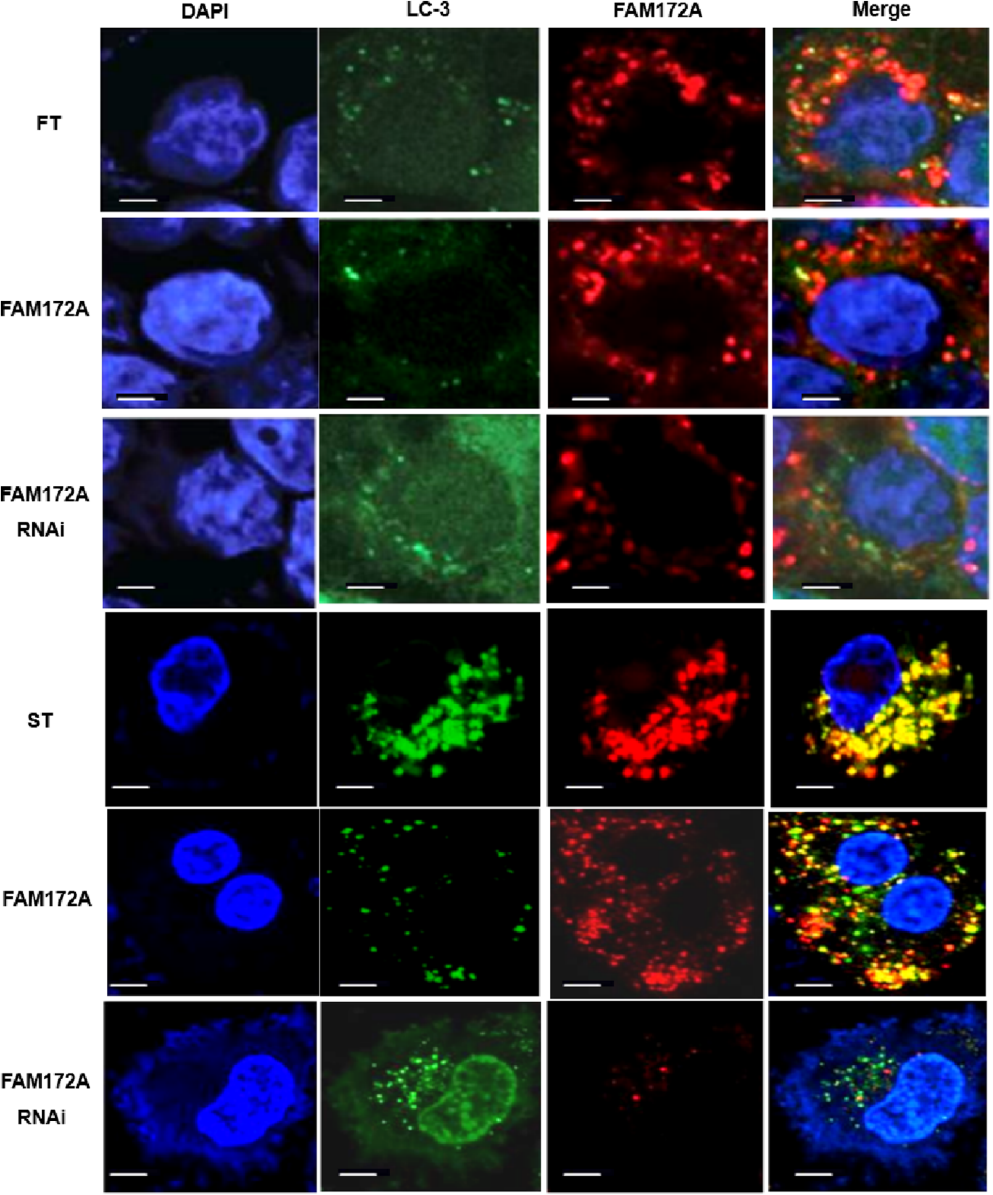
The application of confocal images confirmed that FAM72A participated in the regulation of fibroblasts' autophagy. Further application of laser confocal microscopy was used to detect the autophagy status of cells based on the expression level of LC‐3 in the two cell lines, FT and ST. (The number of experimental replicates was three. Scale bar was 0.25 μm.)

Further application of laser confocal microscopy was used to detect the autophagy status of cells; the results suggested that FAM172A overexpression restrained the autophagy process of FT and ST, and the autophagy status of ST was more distinct (Figure 8). However, through prohibiting, the expression of FAM172A by interference methods promoted the autophagy process of these two cell lines (Figure 8).

### 3.5 FAM172A suppressed EF associated with the apoptotic autophagy process of fibroblasts

**FIGURE 9:**
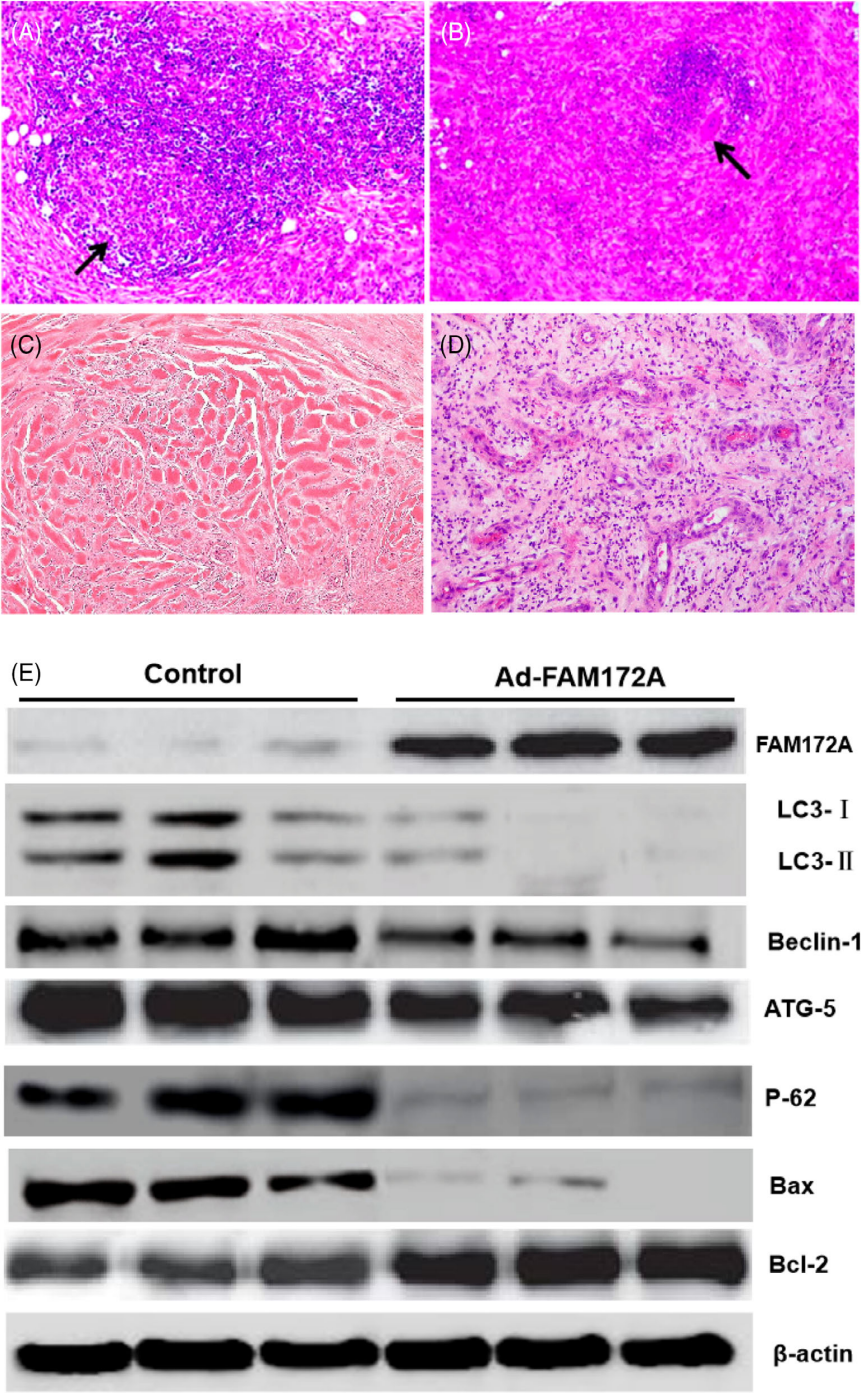
FAM172A suppressed EF associated with the apoptotic autophagy process in fibroblasts. Based on the mice model, we conducted overexpression of FAM172A in the EF process to identify its role associated with the apoptotic autophagy of fibroblasts. The HE staining was used to show typical features of scar tissues (A–D; ×200). Moreover, the autophagy marker protein LC‐3 and the expression levels of its subtypes LC‐3I and LC‐3II were examined. In the meantime, the expression of FAM172A, the apoptosis‐promoting protein Bax, the apoptosis‐inhibiting protein Bcl‐2, the key regulatory protein complexes of autophagy Beclin‐1, ATG‐5, and autophagy protein p62, were evaluated in tissues of mice with WB method (E). (The number of experimental replicates was three.)

Based on the mice model, we found that overexpression of FAM172A could induce suppression in the process of EF information. The HE images of the control group showed typical features of scar tissues (Figures 9A and C). A large number of parallel or staggered collagen fiber bundles could be seen in scar tissues. The fiber bundles were homogeneously red stained, with glassy change, few fiber cells, long and thin nuclei, and deep staining, and blood vessels were rare. However, in the mice model that dealt with Ad‐FAM172A, the fibrosis and scar tissues were significantly reduced to controls (Figures 9B and D). Our results indicated that FAM172A could restrain EF in the mice model.

Moreover, through examining the autophagy marker protein LC‐3, especially for its subtypes LC‐3I and LC‐3II expression levels, we found that the overexpression of FAM172A resulted in a decreased expression ratio of the autophagy marker protein LC3‐II in laminectomy model mice (Figure 9E).

In the meantime, the overexpression of FAM172A resulted in decreased expression of the key regulatory protein complexes of autophagy Beclin‐1, ATG‐5, and autophagy protein p62. In addition, FAM172A overexpression inhibited the expression level of apoptosis‐promoting protein Bax and promoted the expression of apoptosis‐inhibiting protein Bcl‐2 (Figure 9E and Figure Figure 9E).

## 4 DISCUSSION

With the extensive development of lumbar spine surgery, the incidence of failed back surgery syndrome (FBSS) has increased significantly, which has seriously affected the quality of life (QOL) of patients. Lumbar spine surgery failure syndrome is a common complication of posterior lumbar spine surgery. Not only epidural fibrosis (EF) is the main cause, but also there are no effective treatments for EF at present.^1^ Therefore, exploring its pathogenesis and effective drugs highlights its importance. We thought that the main mechanism of epidural fibrosis is the local proliferation of fibroblasts.

Fibroblasts are critical in supporting normal wound healing, involved in key processes such as breaking down the fibrin clot, creating new extracellular matrix (ECM) and collagen structures to support the other cells associated with effective wound healing, as well as contracting the wound.^28^ But, the abnormal proliferation of fibroblasts may cause the scar formation. The cellular homeostasis of fibroblasts is major maintained by the catabolic pathway of autophagy, which plays an important role in proliferation, and is associated with ER stress.^2^, ^3^, ^4^ Moreover, ER stress was related to the cytosolic changes in the concentrations of calcium ions.^17^, ^18^

In previous work, it was found that the potent‐binding activity between FAM172A protein and Ca^2+^ ion and the significantly downregulated expression of FAM172A in ER stress situation induced by tunicamycin.^14^ FAM172A was the key factor for the cytosolic changes of Ca^2+^ concentrations,^14^, ^15^ which was related to ER stress. However, the intracellular calcium signaling pathways are referred to the variety of biological processes in cells, such as growth, apoptosis, differentiation, autophagy, and metastasis.^29^ Therefore, the in‐depth and comprehensive research involved in the interaction mechanism between apoptosis and autophagy must take breakthroughs on the cognition and treatment of diseases, such as scar formation and epidural fibrosis.^30^ Additionally, in some cell lines, the influx of calcium monitors the ER stress.^31^

Consequently, we successfully induced the endoplasmic reticulum (ER) stress with tunicamycin in fibroblasts, the two cell lines come from normal fiber tissue (FT) and epidural scar tissue (ST), respectively. Moreover, ER stress was positively correlated to the intracellular calcium flux mediated by FAM172A, especially in ST. CHOP is a homologous protein of the transcription factor C/EBP (CCAAT enhancer‐binding protein) and could inhibit the transcription factor C/EBP and LAP (Liver‐enriched activator protein).^19^ Some cell stress, such as starvation, could induce CHOP expression, and CHOP inhibits cell cycle transition from G1 to S phase.^20^ Recent studies have found that the expression level of CHOP is upregulated under ER stress, and CHOP mediates programmed cell death or apoptosis. CHOP can mediate the activation of GADD 34 during the endoplasmic reticulum stress.^21^ In this work, we confirmed that CHOP showed a dose‐dependent increase, followed by the increased concentration of the tunicamycin, especially in ST. Hence, our results indicated that the calcium current in ST was significantly stronger than that in FT tissue. In a word, we confirmed that tunicamycin‐induced ER stress in fibroblasts is associated with calcium flux mediated by FAM172A.

The higher organisms all need the essential calcium for survival; the dynamic 2nd messenger calcium is versatile.^32^ The endoplasmic reticulum releases calcium under excitation and activation of cells, then phosphatases and kinases that depended on calcium are activated, meanwhile numerous cellular processes are regulated, including autophagy and apoptosis.^33^ Several identified regulated mechanisms of autophagy are related to calcium ions, meanwhile important to maintain cell survival and mediate life and death decisions.^32^

Furthermore, through up‐ or downregulation of FAM172A expression, we explored whether FAM172A regulates the process of ER stress and the calcium flux in the two fibrosis cell lines. Consequently, we figured out that FAM72A participated in the regulation of the endoplasmic reticulum stress‐related calcium flux in a negative role. In brief, the downregulation of FAM172A expression maybe the key pathogenic factor of the epidural fibrosis process, and it was mediated by the process of the endoplasmic reticulum stress‐related calcium flux. Moreover, it was inconsistent with the previous study,^34^ which results suggested that PPP1R13B regulated by circ‐012091 promoted the proliferation and migration of lung fibroblasts through regulation of ERS and autophagy and played a crucial role in the development of pulmonary fibrosis in silicosis.^34^

According to the different regulation methods of apoptosis and autophagy, the interaction can be roughly summarized into three types, including cooperative relationship, confrontation relationship, and promotion relationship.^35^, ^36^, ^37^, ^38^, ^39^ The multiple interaction modes between autophagy and apoptosis must have common signaling pathways and regulatory proteins, which researchers call interaction regulators.^40^ Wan et al. validated that the proliferation of fibroblasts was considered to be a major cause in the formation process of epidural fibrosis. Artesunate could inhibit the proliferation of fibroblasts and reduce the formation of epidural fibrosis after laminectomy. Its potential mechanism might be associated with the autophagy cascade mediated by p53/p21^waf1/cip1^ pathway. Artesunate might provide a novel reagent for reducing epidural fibrosis after spinal laminectomy surgery.^41^ Additionally, Dai et al. provided also that a traditional Chinese medicine, triptolide (TP), could reduce the formation of epidural fibrosis, and the potential mechanism might inhibit the proliferation of fibroblasts or cause apoptosis and autophagy via suppressing PI3K/AKT/mTOR signaling pathway. It might provide a novel thought for reducing surgery‐induced epidural fibrosis.^42^

Therefore, the cell lines come from normal fibrosis tissue (FT), and epidural scar tissue (ST) was further evolved autophagy induced by tunicamycin, meanwhile cellular apoptotic progression was suppressed, especially in ST. It confirmed that this apoptotic autophagy was regulated by FAM172A in ST. So, we thought that FAM72A participated in regulating the autophagy of fibrosis cells. The multiple interaction modes of apoptosis and autophagy in fibrosis cells, especially for epidural scar cells, must have the common signaling pathway and regulatory protein involved in FAM172A, which maybe the key interaction regulator.

## 5 CONCLUSIONS

Tunicamycin‐induced endoplasmic reticulum (ER) stress in fibroblasts was associated with calcium flux mediated by FAM172A. FAM72A participated in the autophagy regulation of fibroblasts. FAM172A maybe the key interaction regulator of apoptosis and autophagy in fibroblasts, especially for epidural scar cells.

## CONSENT FOR PUBLICATION

Not applicable.

## AVAILABILITY OF DATA AND MATERIALS

The data used to support the findings of this study are included within the article.

## COMPETING INTERESTS

The authors declare that they have no competing interests.

## Supporting information


Appendix S1
