# Supplementary material for: FAM172A supervises ER (endoplasmic reticulum) stress‐triggered autophagy in the epidural fibrosis process
Source: JOR Spine. 2022 May 1;5(2):e1203. doi: 10.1002/jsp2.1203 (PMC9238286; doi:10.1002/jsp2.1203)
Supplement: Supplementary file 1 — Appendix S1 [file JSP2-5-e1203-s001.doc]

***Supplementary figures:***

***Kruskal-Wallis ANOVA (Analysis of variance) was used to analyzed these data among groups. SPSS software (version 18.0) was utilized to perform these statistical analyses. P<0.05 was significant statistically.***


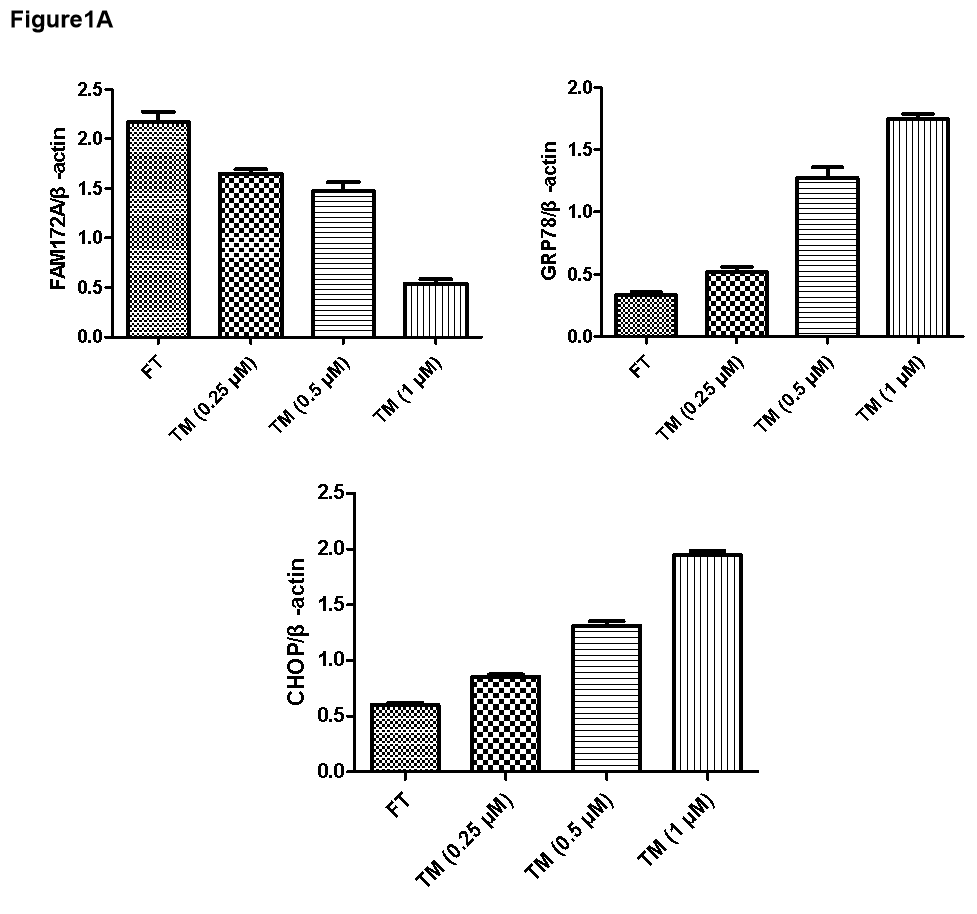


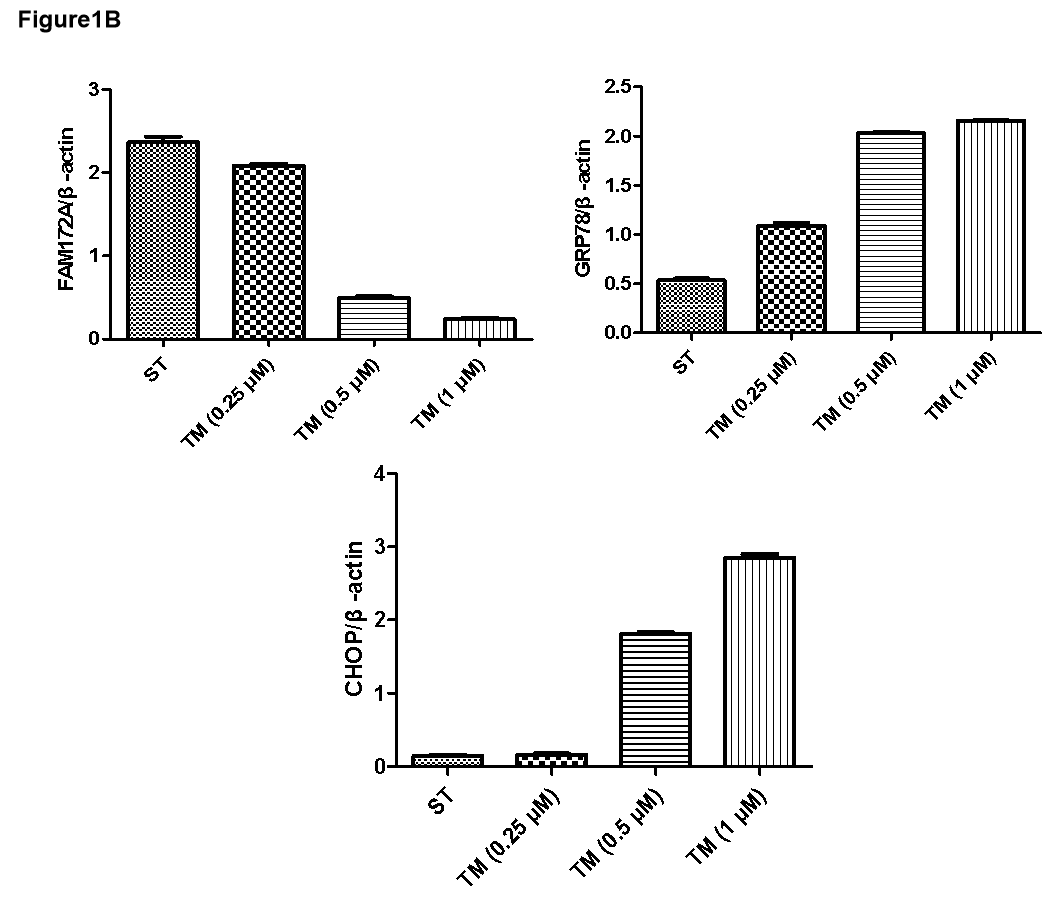


**Supplementary Figure 1. Tunicamycin-induced the endoplasmic reticulum (ER) stress in fibroblasts was associated with calcuim flux regulated by FAM172A.**

At first, we used the recognized tunicamycin to induce the endoplasmic reticulum (ER) stress in fibroblasts. After treatment with tunicamycin, FT (Fibrosis tissue) (A) and ST (Epidural scar tissue) (B) both evolved into the endoplasmic reticulum (ER) stress. The expression of endoplasmic reticulum stress marker protein GRP78 showed a dose-dependent increase with the raising concentration of tunicamycin, especially in ST. On the contrary, the expression of FAM172A showed a dose-dependent decrease with the stepwise tunicamycin concentration, and it was more significant in ST.

Our experimental data indicated that the key protein of the endoplasmic reticulum stress signaling pathway, CHOP also increased in a dose-dependent manner with the stepwise tunicamycin concentration, and its expression was more significant in FT (A and B).


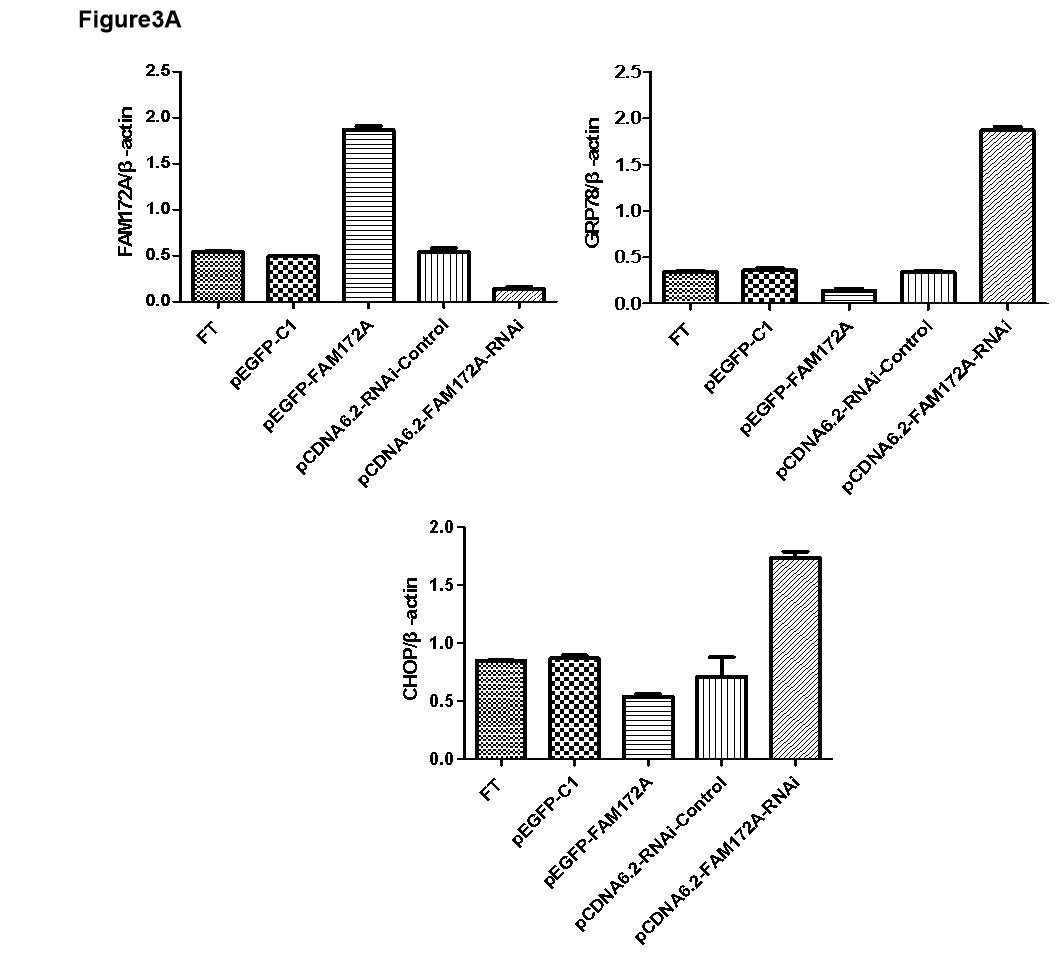


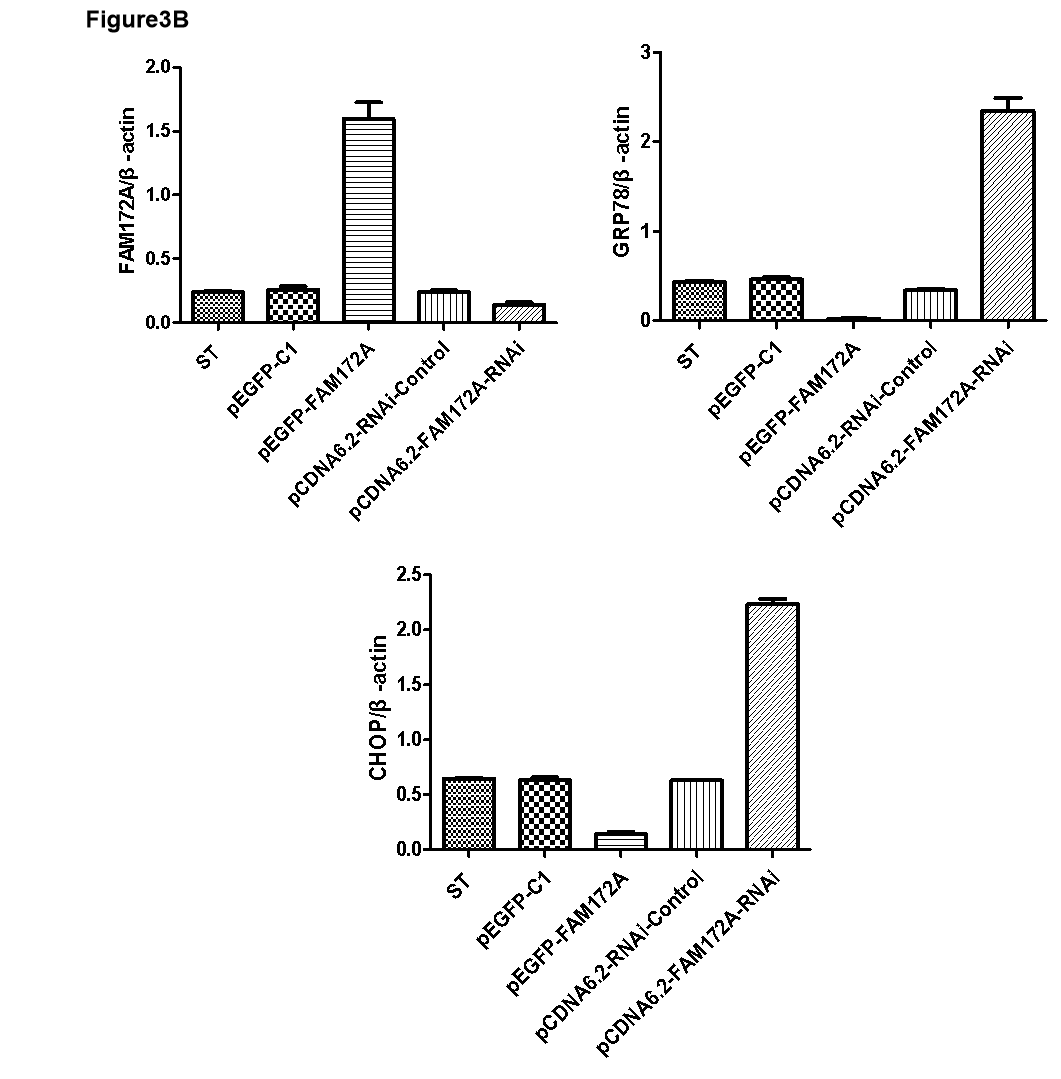


**Supplementary Figure 3. FAM72A participated in the regulation of the endoplasmic reticulum stress-related calcuim flux.**

Through over-expression and interference with the expression of FAM172A, it was aimed to clear whether FAM172A regulated the process of the endoplasmic reticulum stress in FT (A) and ST (B). By examining the expression levels of the marker protein GRP78 of endoplasmic reticulum stress and the key protein CHOP in signal transduction pathway of the ER stress, we found that the over-expression of FAM172A resulted in a decreased expression levels of GRP78 and CHOP in the two cell lines, and ST was more obviously. On the contrary, the interference with the expression of FAM172A caused a significant increased expression levels of GRP78 and CHOP in the two cell lines, and the epidural scar tissue cell line ST was more obvious (A and B).


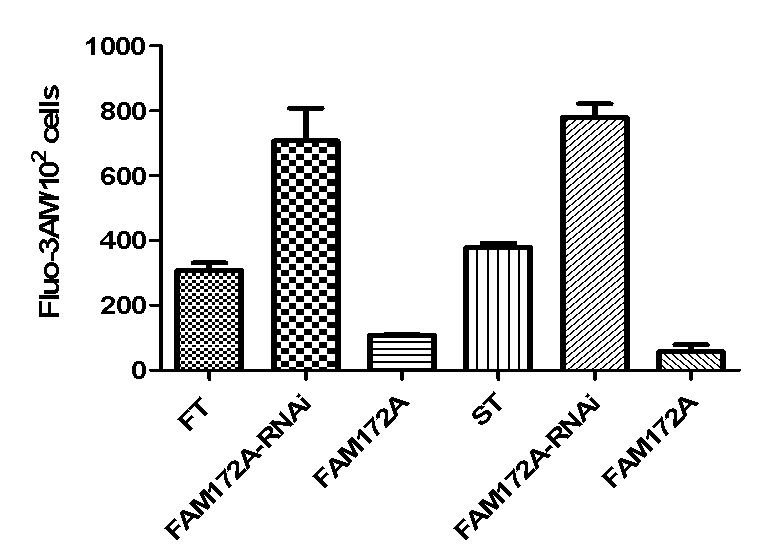
**Supplementary Figure 4. FAM72A participated in the regulation of the endoplasmic reticulum stress-related calcuim flux.**

The laser confocal microscope was further used to detect intracellular calcuim flux, meanwhile calreticulin was used as an internal reference. The results suggested that interference with FAM172A suppressed its expression resulted in a evident increase of intracellular calcuim flux (*P*<0.01), and the epidural scar tissue cell line ST was more visible. On the contrary, over-expression of FAM172A significantly prohibited intracellular calcuim flux (P<0.01), especially in the epidural scar tissue cell line ST.


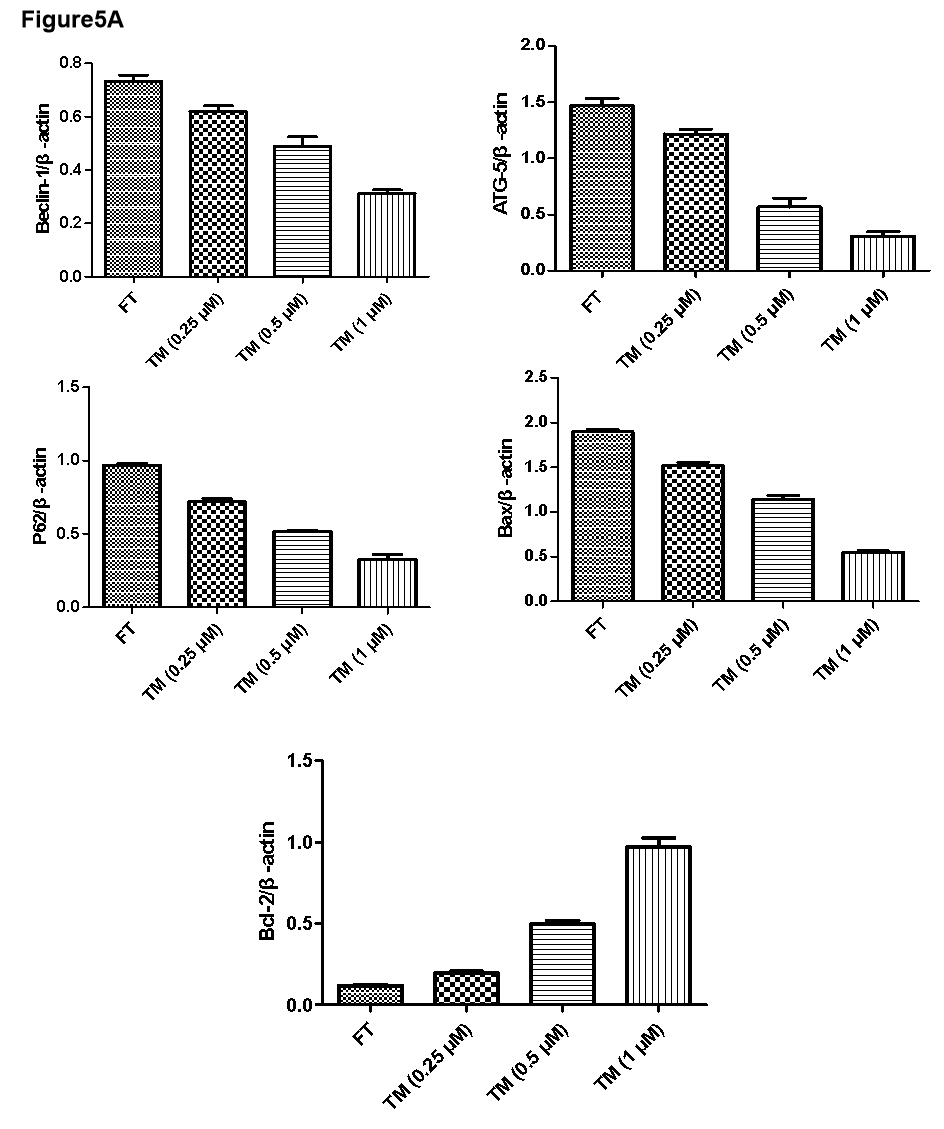

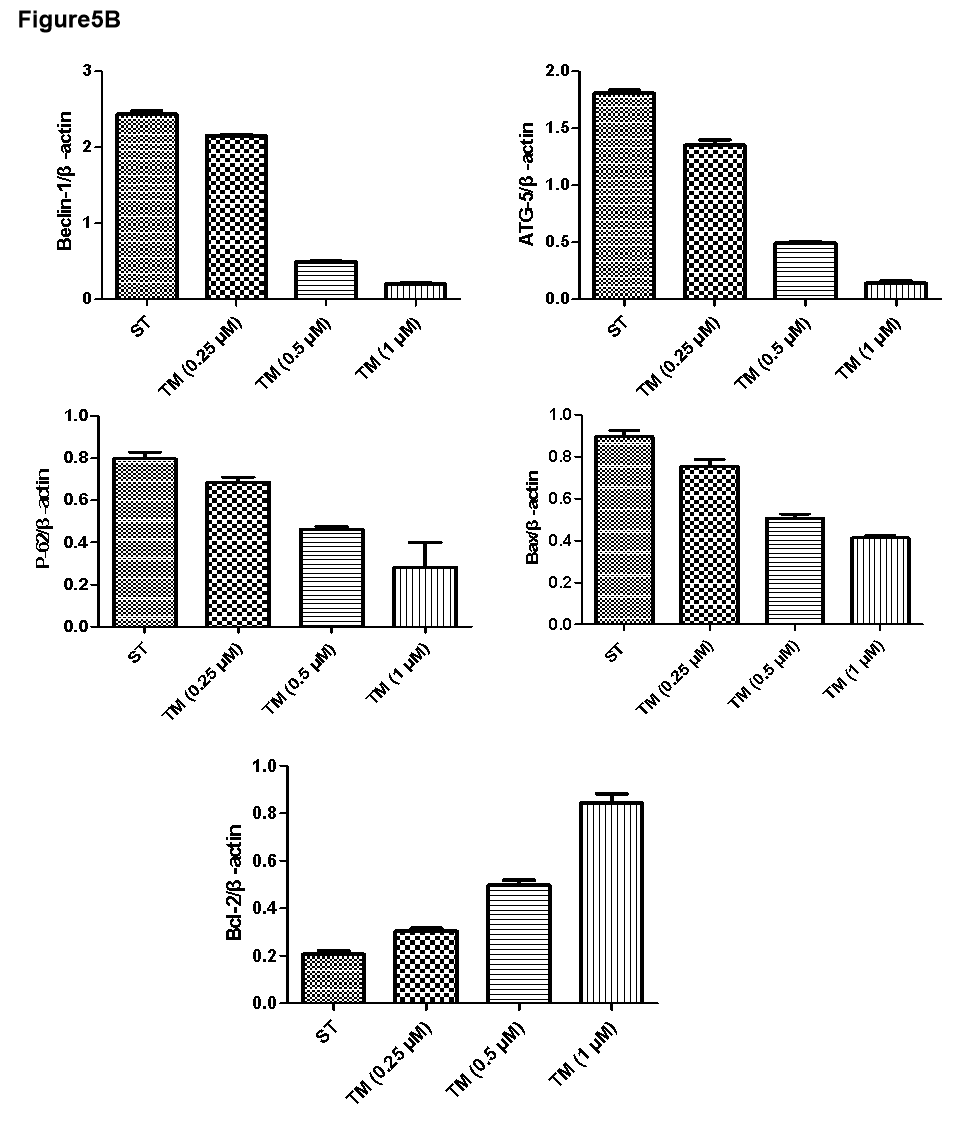
**Supplemenatry Figure 5. Tunicamycin induced autophagy of cell lines and inhibited cell apoptosis regulated by FAM172A.**

After tunicamycin treatment, both the two cell lines, FT (A) and ST (B) occurred autophagy (A and B). Besides, the expression of autophagy key regulatory protein Beclin-1, ATG-5 and autophagy protein p62 all showed a dose-dependent decline followed with the increased tunicamycin concentration, and the expression of these marker proteins in epidural scar tissue cell line ST were more obviously decreased. On the contrary, the expression of apoptosis-promoting protein Bax of the Bcl-2 family, also declined in a dose-dependent manner as the enhanced concentration of tunicamycin. Moreover, the expression of the apoptosis-inhibiting protein Bcl-2 increased significantly, and in the epidural scar tissue cell line ST was more prominent.


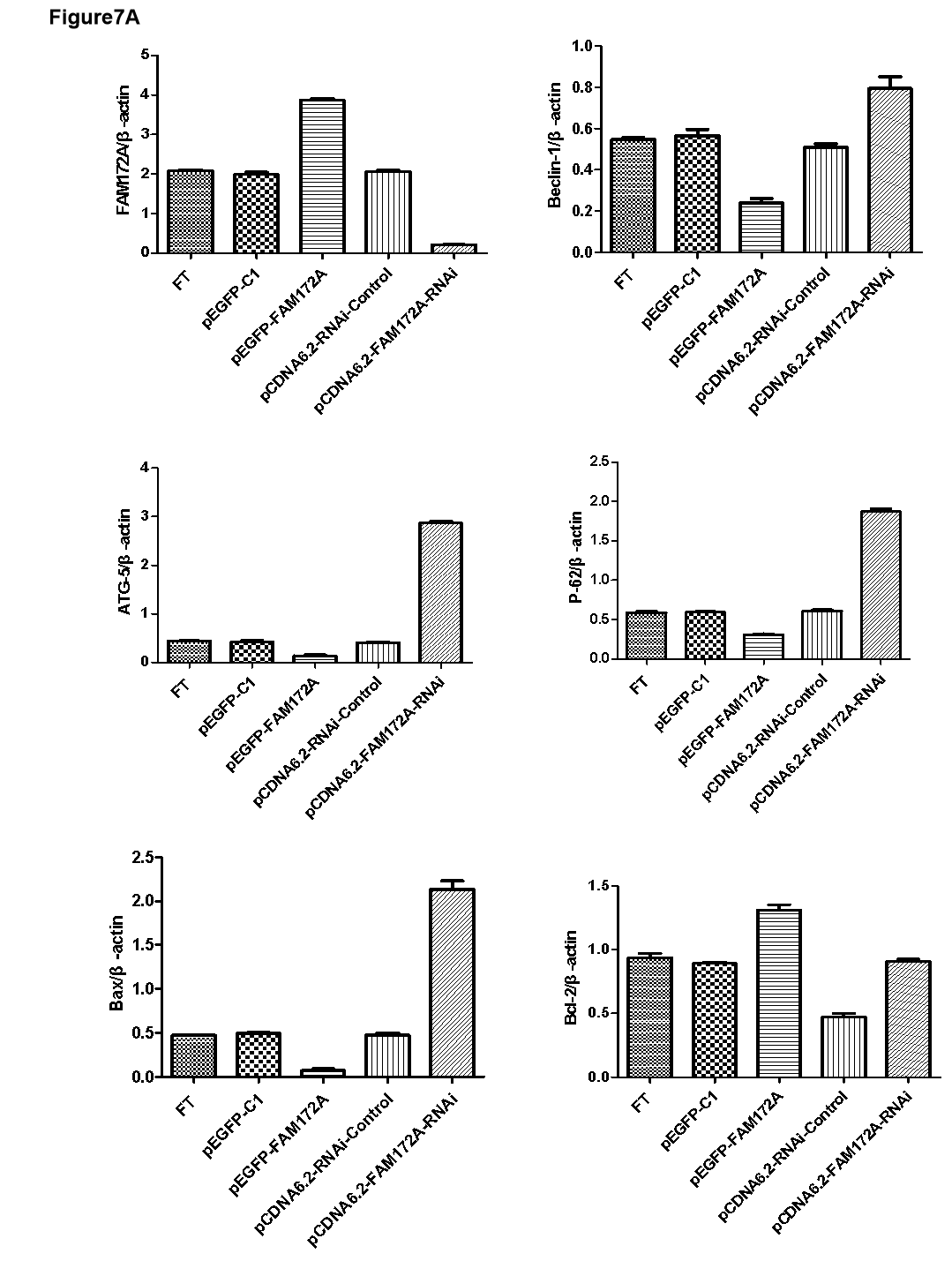


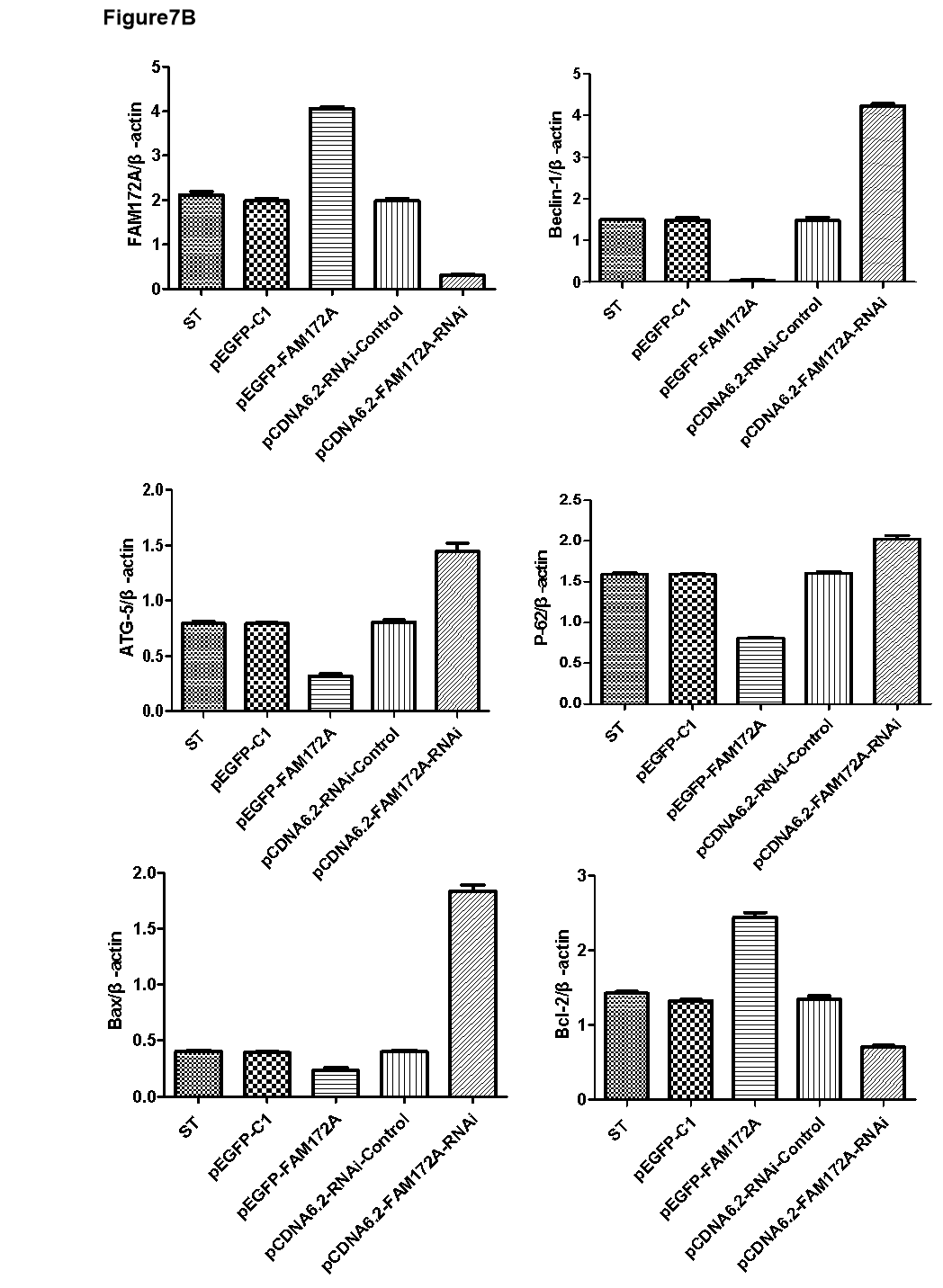


**Supplemenatry Figure 7. FAM72A participated in the regulation apoptotic autophagy process of fibroblasts.**

Through over-expression and interference with the expression of FAM172A, the purpose was to confirm whether FAM172A regulated the autophagy process of FT (A) and ST (B). Through examining the autophagy marker protein LC-3, especially for the expression levels of its subtypes LC-3Ⅰ and LC-3Ⅱ, we found that the over-expression of FAM172A resulted in a decreased expression ratio of the autophagy marker protein LC3-Ⅱ in the two cell lines, in the meantime the epidural scar tissue cell line ST was more prominent. On the contrary, interference with the expression of FAM172A made for a visibly increased expression ratio of the autophagy marker protein LC3-II in the two cell lines, while the normal fibrosis tissue cell line was more evident at this time.

Besides, the over-expression of FAM172A resulted in decreased expression of the key regulatory protein complexes of autophagy Beclin-1, ATG-5 and autophagy protein p62 in the two cell lines, and this was also more pronounced in ST. In addition, FAM172A over-expression inhibited the expression level of apoptosis-promoting protein Bax, and promoted the expression of apoptosis-inhibiting protein Bcl-2, this was more marked in the ST.


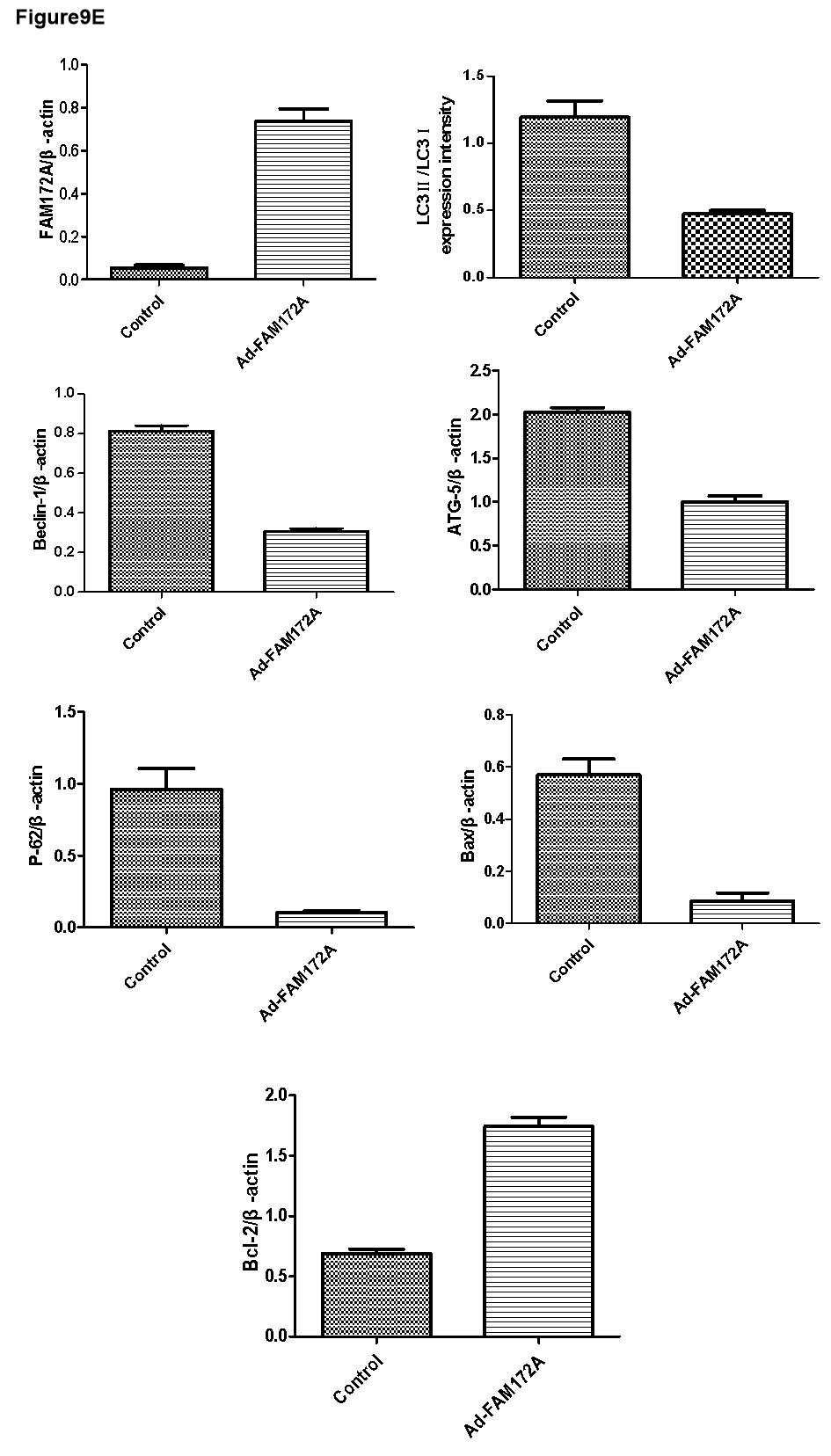


**Supplemenatry Figure 9. FAM172A suppressed EF associated wiht the apoptotic autophagy process of fibroblasts.**

Moreover, through examining the autophagy marker protein LC-3, especially for its subtypes LC-3Ⅰ and LC-3Ⅱ expression levels, we found that the over-expression of FAM172A resulted in a decreased expression ratio of the autophagy marker protein LC3-Ⅱ in laminectomy model mice.

At the meantime, the over-expression of FAM172A resulted in decreased expression of the key regulatory protein complexes of autophagy Beclin-1, ATG-5 and autophagy protein p62. In addition, FAM172A over-expression inhibited the expression level of apoptosis-promoting protein Bax, and promoted the expression of apoptosis-inhibiting protein Bcl-2.
